# The Impact of Diabetes on Outcomes in Anterior Cervical Discectomy and Fusion (ACDF)

**DOI:** 10.3390/jcm14093039

**Published:** 2025-04-28

**Authors:** David Maman, Assil Mahamid, Gabriel Nisanov, Oluwaseun Fagbamila, Ali Sleiman, Arsen Shpigelman, Yaron Berkovich

**Affiliations:** 1 Carmel Medical Center, Haifa 3436212, Israelarsen4ik@gmail.com (A.S.); yaron.berkovich@gmail.com (Y.B.); 2 Faculty of Medicine, Technion Israel Institute of Technology, Haifa 2611001, Israel; gabnis3@gmail.com; 3 Hillel Yaffe Medical Center, Hadera 3810101, Israel; dr.assilm@gmail.com; 4 Faculty of Medicine, Saint James School of Medicine, Kingstown VC0100, Saint Vincent and the Grenadines; seun.f.fagbamila@gmail.com

**Keywords:** anterior cervical discectomy and fusion, type 2 diabetes, postoperative complications, length of stay, healthcare costs

## Abstract

**Background:** Anterior cervical discectomy and fusion (ACDF) is a common treatment for cervical radiculopathy and myelopathy. While generally effective, diabetes mellitus may increase postoperative complications and healthcare costs. This study evaluated the impact of type 2 diabetes on perioperative outcomes in ACDF patients. **Methods:** A retrospective cohort study was conducted using the Nationwide Inpatient Sample (2016–2019), including 85,585 single-level ACDF patients. Propensity score matching (PSM) was applied, creating two balanced cohorts (16,260 diabetic and 16,260 non-diabetic patients). Outcomes analyzed included postoperative complications, length of stay, hospital charges, and mortality. **Results:** Diabetic patients had significantly higher risks of ACDF-specific complications, including cerebrospinal fluid leaks (2×), dysphagia (2.5×), dysphonia (2.9×), and cervical spinal cord injury (5×). General complications were also increased, with higher rates of pulmonary embolism (2.4×), sepsis (3×), stroke (3×), pneumonia (3.3×), and heart failure (12×). Diabetic patients had longer hospital stays (1.99 vs. 1.79 days, *p* < 0.001) and higher hospital charges (USD 71,884 vs. USD 67,998, *p* = 0.004). **Conclusions:** T2DM significantly increases postoperative risks, length of stay, and costs for ACDF patients. Optimized perioperative management and glucose control are essential to improve outcomes in this high-risk population.

## 1. Introduction

Anterior cervical discectomy and fusion (ACDF) is the gold standard for treating cervical radiculopathy and myelopathy, particularly in cases of degenerative spine disease such as spondylosis and disc herniation [1]. As the global population ages, the prevalence of these conditions is expected to increase, leading to a higher demand for ACDF [2]. While the procedure is associated with high success rates and favorable long-term outcomes, patient-specific factors, including diabetes mellitus, can significantly influence postoperative recovery and complication rates [3].

T2DM is a well-established risk factor for poor surgical outcomes due to its association with microvascular dysfunction, impaired wound healing, and an increased susceptibility to infection [4]. These mechanisms may impair spinal fusion through reduced angiogenesis and osteogenesis at the graft site, ultimately delaying or compromising fusion integrity. These physiological changes can hinder bone fusion, delay recovery, and elevate the risk of complications after ACDF. The World Health Organization (WHO) defines T2DM based on fasting plasma glucose, 2 h post-load glucose, HbA1c, or random plasma glucose levels, with disease severity categorized based on glycemic control and the presence of diabetes-related complications [5,6]. With over 800 million individuals affected globally, T2DM is an increasingly important consideration in perioperative risk assessment, particularly in spine surgery [7].

Despite previous studies linking T2DM to increased morbidity in spinal procedures, the specific impact of T2DM severity on ACDF outcomes remains unclear [8]. Understanding whether T2DM contributes to longer hospital stays, higher complication rates, and increased healthcare costs is essential for optimizing perioperative management and improving patient selection [9,10]. This study aimed to assess the prevalence of T2DM among ACDF patients and determine whether the presence of T2DM affects postoperative outcomes, providing valuable insights to refine clinical decision-making and risk stratification.

This analysis examined the relationship between T2DM and ACDF outcomes, focusing on its prevalence, impact on complications, hospital stay, and healthcare costs. Findings will help improve patient selection and perioperative management strategies, ultimately enhancing surgical outcomes.

### Research Questions

This study explored how T2DM influences ACDF outcomes by examining its prevalence, impact on complications, hospital stay, and costs. Findings will help refine patient selection and perioperative management strategies.

## 2. Methods

### 2.1. Dataset Acquisition

This study utilized a dataset extracted from the Nationwide Inpatient Sample (NIS), the largest publicly available all-payer inpatient care database in the United States. A total of 85,585 patients undergoing ACDF were included in the analysis, consisting of 69,325 patients without T2DM and 16,260 patients with type II diabetes.

### 2.2. Study Period and Data Source

Data was collected from 1 January 2016, to 31 December 2019, from the NIS. The NIS database, part of the Healthcare Cost and Utilization Project (HCUP), captures approximately 20% of inpatient stays from HCUP-associated hospitals, covering over seven million unweighted enrollments annually. This dataset represents the most up-to-date pre-COVID-19 period, avoiding the potential biases introduced by the pandemic, which significantly impacted surgical outcomes, hospital resource allocation, and patient management.

### 2.3. Patient Identification and Exclusions

Patients undergoing single-level ACDF were identified using ICD-10 procedure codes. Specific ICD-10-PCS codes used to identify ACDF procedures are listed in Supplementary Table S1. Exclusions included patients under the age of 18, non-elective admissions, and those with revision surgeries or concurrent complex spine procedures. To ensure inclusion of only single-level ACDF, patients undergoing multilevel fusion (n = 21,842) were excluded based on the presence of additional fusion codes or multiple vertebral site indicators. Patients with T2DM were identified using ICD-10-CM diagnosis codes (see Supplementary Table S1 for codes).

### 2.4. Statistical Analyses and Propensity Score Matching

Statistical analyses were conducted using SPSS 26 for descriptive and inferential statistics, while MATLAB 2024 was used for propensity score matching and visualization. This combination allowed for robust statistical computing and flexible matching algorithms not available in SPSS. Crosstabs and independent sample *t*-tests were performed to compare patients with and without type II diabetes, with statistical significance set at *p* < 0.05.

To control confounding factors, a propensity score matching (PSM) analysis was performed using MATLAB. Matching variables included demographic data (age, gender, and payer status), comorbidities, and hospital characteristics. These were selected based on clinical relevance and prior literature indicating their influence on postoperative outcomes in spine surgery. The matching process resulted in two cohorts of 16,260 patients each, with balanced baseline characteristics to minimize selection bias. PSM was performed using 1:1 nearest-neighbor matching without replacement, with a caliper width of 0.2 standard deviations of the logit of the propensity score. Matching was based on variables including age, gender, primary payer, hospital region, and comorbidities. Standardized mean differences (SMDs) for all covariates before and after matching were calculated to confirm covariate balance.

### 2.5. Comorbidity and Outcome Identification

Comorbidities and postoperative complications were identified based on ICD-10 codes. Outcomes of interest included in-hospital mortality, length of stay, total hospital charges, and both general postoperative complications (e.g., urinary tract infections, blood loss anemia, and deep vein thrombosis) and specific postoperative complications related to ACDF (e.g., dysphagia, Horner syndrome, and cervical spinal cord injury). Operational definitions for each outcome were based on ICD-10-CM diagnosis codes recorded during the index hospitalization, and are provided in Supplementary Table S2.

### 2.6. Risk Ratio Analysis

Risk ratio (RR) analyses were conducted to evaluate the relative risks of both general and ACDF-specific postoperative complications in patients with T2DM compared to non-diabetic patients. Risk ratios were calculated along with 95% confidence intervals (CI) for each complication. This approach provided a comprehensive assessment of the relative likelihood of adverse outcomes associated with type II diabetes.

### 2.7. Ethical Considerations

This study was a retrospective cohort analysis utilizing de-identified data from the NIS database and was exempt from institutional review board approval. Informed consent was not required, as the NIS database does not include patient-identifiable information.

## 3. Results

### 3.1. Demographics and Insurance Characteristics

Table 1 provides a comparison of demographic and insurance characteristics between patients undergoing ACDF with and without type II diabetes.

Patients with T2DM were significantly older and there was a lower proportion of female patients (47.0% vs. 52.8%, *p* < 0.001) compared to non-diabetic patients.

Significant differences were also observed in the primary expected payer categories. Medicare coverage was substantially more common among patients with T2DM (47.8% vs. 30.6%, *p* < 0.001), reflecting the older age of this cohort. Conversely, private insurance coverage was more prevalent among non-diabetic patients (46.9% vs. 34%, *p* < 0.001). Across the study period, annual case volumes were relatively consistent, with 20,726 cases in 2016, 21,347 in 2017, 21,582 in 2018, and 21,930 in 2019, totaling 85,585 single-level ACDF procedures.

### 3.2. Comparative Analysis of Comorbidities in ACDF Patients with and Without Type II Diabetes

Table 2 provides an analysis of comorbidities in patients undergoing ACDF, comparing those with and without type II diabetes. These results reveal that T2DM is associated with a significantly higher prevalence of multiple chronic conditions.

Patients with T2DM had notably higher rates of hypertension and chronic kidney disease. The prevalence of obstructive sleep apnea and obesity was also significantly greater in the diabetic cohort. In contrast, no significant difference was observed in the prevalence of alcohol abuse or fibromyalgia.

### 3.3. Propensity Score-Matched Analysis

To address potential selection bias and baseline differences in demographic and comorbidity variables, a propensity score-matched analysis was conducted. This method matched individuals based on their likelihood of belonging to either group (with or without type II diabetes), and ensured statistically equivalent groups for comparison. By balancing baseline characteristics, this approach minimized confounding factors and improved the reliability of these results, mimicking the randomization process in experimental studies.

Table 3 provides a detailed comparison of demographic characteristics and comorbidities between single-level ACDF patients with and without T2DM after propensity score matching. Each group included 16,260 patients, and the results demonstrated no statistically significant differences in age, gender distribution, primary expected payer, or most comorbidities.

These findings confirm the effectiveness of the propensity score-matching process, as both groups showed near-identical distributions for variables, ensuring fair comparisons between patients with and without T2DM undergoing single-level ACDF.

### 3.4. Comparison of Hospitalization Outcomes in Propensity Score-Matched Single-Level ACDF Patients

Hospitalization outcomes were evaluated in propensity score-matched cohorts of single-level ACDF patients with and without T2DM, as shown in Table 4. Patients with T2DM had a longer average length of stay (1.99 days, Std. deviation 2.6) compared to those without T2DM (1.79 days, Std. deviation 2.3, *p* < 0.001). Total hospital charges were higher for diabetic patients (USD 71,884, Std. deviation USD 55,528) than for non-diabetic patients (USD 67,998, Std. deviation USD 56,191, *p* = 0.004).

### 3.5. Comparison of Select Postoperative Complications in Propensity Score-Matched Single-Level ACDF Patients

Table 5 presents a comparison of select postoperative complications in propensity score-matched cohorts of single-level ACDF patients with and without type II diabetes. These complications showed no statistically significant differences. DVT and blood loss anemia were selected as representative general complications commonly analyzed in the spinal surgery literature and NIS-based studies. These reflect clinically relevant and frequently coded events.

### 3.6. Elevated Risk Ratios for General Postoperative Complications in Single-Level ACDF Patients with Type II Diabetes

**Figure 1:**
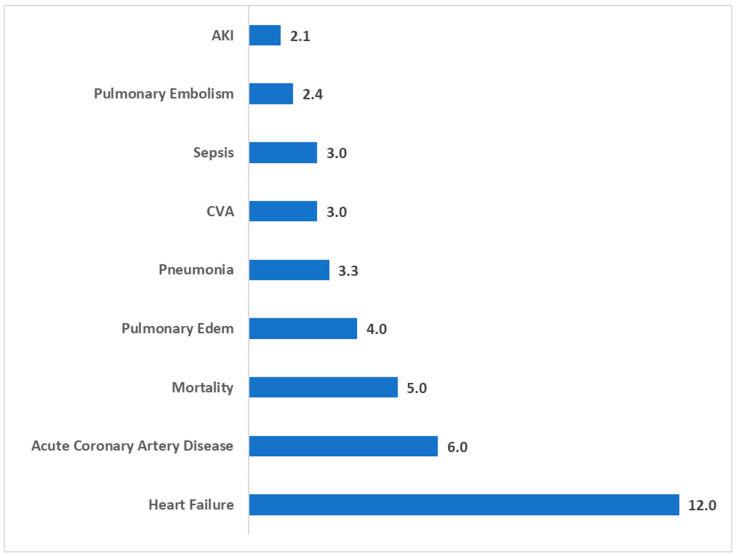
Risk ratios for general postoperative complications in single-level ACDF patients with type II diabetes compared to non-diabetic patients.

Figure 1 illustrates the RR for general postoperative complications in single-level ACDF patients with T2DM compared to propensity score-matched non-diabetic patients. These findings reveal significantly elevated risks for a range of complications. Heart failure exhibited the highest risk, with a risk ratio of 12.0 (95% CI: 4.8–29.9, *p* < 0.001), followed by acute coronary artery disease with a risk ratio of 6.0 (95% CI: 2.3–15.5, *p* < 0.001). Mortality had a risk ratio of 5.0 (95% CI: 1.9–13.1, *p* < 0.001).

Other significant complications included pulmonary edema, with a risk ratio of 4.0 (95% CI: 1.5–10.7, *p* = 0.003), and pneumonia, with a risk ratio of 3.3 (95% CI: 2.0–5.4, *p* < 0.001). Cerebrovascular accident (CVA) and sepsis were also more common in diabetic patients, with risk ratios of 3.0 (95% CI: 1.9–8.2, *p* = 0.025) and 3.0 (95% CI: 1.7–5.4, *p* < 0.001), respectively. Pulmonary embolism showed a risk ratio of 2.4 (95% CI: 1.3–4.3, *p* = 0.005). Finally, acute kidney injury (AKI) had a significantly elevated risk ratio of 2.1 (95% CI: 1.65–2.6, *p* < 0.001).

### 3.7. Elevated Risk Ratios for ACDF-Specific Complications in Single-Level ACDF Patients with Type II Diabetes

**Figure 2:**
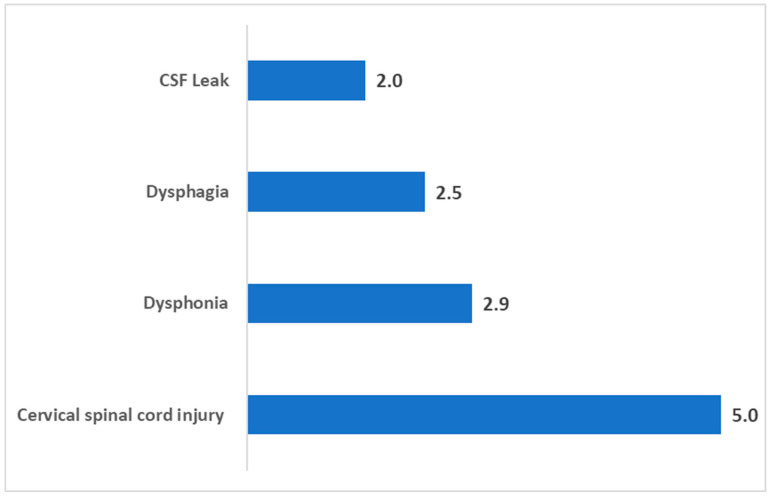
Risk ratios for ACDF-specific complications in single-level ACDF patients with type II diabetes compared to non-diabetic patients.

Figure 2 highlights risk ratios for ACDF-specific complications in single-level ACDF patients with T2DM compared to propensity score-matched non-diabetic patients. These findings reveal significantly elevated risks for complications unique to the procedure. Cervical spinal cord injury showed the highest risk, with a risk ratio of 5.0 (95% CI: 2.5–9.9, *p* < 0.001). Dysphonia was also significantly more common in diabetic patients, with a risk ratio of 2.9 (95% CI: 1.9–4.2, *p* < 0.001). Dysphagia exhibited a risk ratio of 2.5 (95% CI: 2.2–2.8, *p* < 0.001), and CSF leak demonstrated an elevated risk ratio of 2.0 (95% CI: 1.2–3.4, *p* = 0.010).

## 4. Discussion

In this large, propensity score-matched analysis of over 85,000 patients undergoing single-level ACDF, we found that patients with type 2 diabetes mellitus (T2DM) experienced significantly higher rates of both general and ACDF-specific postoperative complications, longer hospital stays, and increased hospital charges compared to non-diabetic patients. These differences remained statistically significant even after adjusting for baseline demographics, comorbidities, and hospital characteristics using rigorous 1:1 nearest-neighbor matching. This suggests that T2DM may independently contribute to adverse outcomes following ACDF, beyond known confounders.

Our findings are consistent with prior studies demonstrating that patients with T2DM are at an increased risk of complications after spinal fusion procedures [11,12,13]. However, many earlier investigations either grouped various spine surgeries together or lacked comprehensive risk adjustment. In contrast, our study focused exclusively on single-level ACDF procedures and employed strict propensity score matching to create comparable groups. This methodological strength supports the robustness of our findings and helps isolate the impact of diabetes on ACDF-specific outcomes.

We observed particularly elevated risks of cardiovascular and infectious complications among diabetic patients. For example, the relative risk of heart failure was 12.0, of acute coronary artery disease was 6.0, and of sepsis was 3.0. In terms of ACDF-specific complications, patients with T2DM were five times more likely to suffer a cervical spinal cord injury, nearly three times more likely to develop dysphonia, and two-and-a-half times more likely to experience dysphagia. These complications may be attributable to the chronic microvascular dysfunction, impaired immune response, and delayed wound healing associated with diabetes [4,14]. These mechanisms compromise both soft tissue recovery and spinal fusion integrity and have been previously documented as contributing factors to postoperative morbidity in spine surgery [15,16,17].

Interestingly, we found that acute kidney injury (AKI) was slightly more common in non-diabetic patients, despite the known renal vulnerability of T2DM patients. While statistically significant, the absolute difference was small (1.6% vs. 1.3%, *p* = 0.014), and the clinical significance of this finding remains uncertain. Further investigation is warranted to determine whether perioperative glucose management or nephroprotective strategies might influence this observation.

The 23% prevalence of diabetes in our cohort exceeded the 11.8–17.3% range reported in earlier studies of ACDF patients [11,12], potentially reflecting the increasing burden of T2DM in the aging surgical population. Our results reinforce prior observations that T2DM is associated with longer hospitalization and increased healthcare costs following spine surgery [13]. The average hospital charges for diabetic patients were approximately USD 3900 higher than for non-diabetic patients. This economic disparity is in line with global data demonstrating that T2DM-related complications—including cardiovascular disease, nephropathy, and infection—substantially increase inpatient resource utilization [18,19,20].

Our study also supports the growing consensus on the importance of preoperative optimization of diabetic patients. Poor glycemic control is linked to increased risks of reoperation, surgical site infection, and mortality [15,16,21,22]. Professional guidelines recommend HbA1c screening before elective spine surgery, with suggested targets below 7.5% [15,23]. Although our dataset did not include HbA1c levels or insulin dependence, future research should aim to stratify risk according to glycemic control. Prior studies have shown that even patients with “controlled” diabetes may remain at elevated risk [24,25], and that HbA1c levels above 6.8% correlate with worse functional recovery and higher reoperation rates [23,25,26,27,28].

The clinical implications of our findings are significant. They suggest that surgeons should consider diabetes as an independent predictor of adverse outcomes in ACDF and advocate for multidisciplinary preoperative protocols that involve endocrinologists, nutritionists, and anesthesiologists. Operationally, this could involve targeting preoperative HbA1c < 7.0%, perioperative insulin management, and close postoperative monitoring for infections or neurologic deterioration.

Our study had several limitations. The NIS database, while large and nationally representative, is subject to coding inaccuracies and lacks long-term follow-up data. Importantly, glycemic control markers such as HbA1c or diabetes duration are not available, which limited our ability to stratify risk by diabetes severity [29,30,31]. In addition, the NIS only captures in-hospital complications and charges; readmissions, outpatient costs, and long-term fusion success were not assessed. Lastly, although we used robust PSM techniques, residual confounding may still be present.

Despite these limitations, our study provides strong evidence that T2DM independently increases the risk of perioperative complications and costs for ACDF patients. Using a large, nationally representative sample and rigorous matching techniques, we added new clarity to the growing body of literature on this important surgical risk factor.

Future research should focus on integrating lab-based glycemic markers into surgical risk calculators, defining glycemic thresholds for safe elective spine surgery, and evaluating the cost-effectiveness of multidisciplinary optimization programs for diabetic patients.

## 5. Conclusions

T2DM significantly increases postoperative complications, hospital stay, and costs in ACDF patients. Even after adjusting for confounders, T2DM remained an independent risk factor for adverse outcomes. Optimizing perioperative glucose control and risk stratification is crucial to improving surgical outcomes. Future research should refine screening protocols and targeted interventions to mitigate these risks.

## Figures and Tables

**Table 1:** Demographic and insurance characteristics of single-level ACDF patients with and without type 2 diabetes.

Parameter	No Type 2 Diabetes	Type 2 Diabetes	Significance
Total Surgeries	69,325	16,260	-
Average Age (y)	54.5	60.3	*p* < 0.001
Female (%)	52.8	47.0	*p* < 0.001
Primary expected payer—Medicare (%)	30.6	47.8	*p* < 0.001
Primary expected payer—Medicaid (%)	11	9.5	
Primary expected payer—private including HMO (%)	46.9	34	
Primary expected payer—self-pay (%)	1.3	1	
Primary expected payer—no charge (%)	0.1	0.1	
Primary expected payer—other (%)	10.1	7.6	

**Table 2:** Prevalence of comorbidities of single-level ACDF patients with and without type II diabetes.

Parameter	No Type 2 Diabetes	Type 2 Diabetes	Significance
Hypertension (%)	38.4	66.3	*p* < 0.001
Dyslipidemia (%)	24.1	55.1	*p* < 0.001
Obstructive Sleep Apnea (%)	7.6	17.9	*p* < 0.001
Chronic Anemia (%)	2.1	3.2	*p* < 0.001
Alcohol Abuse (%)	1.2	1.1	*p* = 0.376
Osteoporosis (%)	2.2	2.8	*p* < 0.001
Chronic Kidney Disease (%)	2.4	9.6	*p* < 0.001
Congestive Heart Failure (%)	0.6	2.2	*p* < 0.001
Liver Disease (%)	0.9	1.9	*p* < 0.001
History of Myocardial Infarction (%)	2.3	5.5	*p* < 0.001
History of Cerebrovascular Accident (%)	3.2	6.9	*p* < 0.001
Chronic Lung Disease (%)	7.2	11.2	*p* < 0.001
Obesity (%)	15.6	30.9	*p* < 0.001
Fibromyalgia (%)	3.8	4	*p* = 0.177

**Table 3:** Comparison of demographic and clinical data in propensity score-matched cohorts of single-level ACDF patients with and without type II diabetes.

Parameter	No Type 2 Diabetes	Type 2 Diabetes	Significance
Total Surgeries (%)	16,260	16,260	-
Average Age (y)	60.2	60.3	0.65
Female (%)	46.4	47.0	*p* = 0.18
Primary expected payer—Medicare (%)	48	47.8	*p* = 0.13
Primary expected payer—Medicaid (%)	8.7	9.5	
Primary expected payer—private and HMO (%)	35.2	34	
Primary expected payer—self-pay (%)	0.4	1	
Primary expected payer—no charge (%)	0.1	0.1	
Primary expected payer—other (%)	7.6	7.6	
Hypertension (%)	66.8	66.3	*p* = 0.24
Dyslipidemia (%)	54.9	55.1	*p* = 0.70
Obstructive Sleep Apnea (%)	17.1	17.9	*p* = 0.24
Chronic Anemia (%)	2.9	3.2	*p* = 0.43
Alcohol Abuse (%)	1.3	1.1	*p* = 0.28
Osteoporosis (%)	2.8	2.8	*p* = 0.87
Chronic Kidney Disease (%)	9	9.6	*p* = 0.20
Congestive Heart Failure (%)	2	2.2	*p* = 0.07
Liver Disease (%)	2.3	1.9	*p* = 0.05
History of Myocardial Infarction (%)	5.6	5.5	*p* = 0.63
History of Cerebrovascular Accident (%)	6.6	6.9	*p* = 0.30
Chronic Lung Disease (%)	11.2	11.2	*p* = 0.82
Obesity (%)	30.2	30.9	*p* = 0.18
Fibromyalgia (%)	3.9	4	*p* = 0.63

**Table 4:** Comparison of hospitalization outcomes in propensity score-matched cohorts of single-level ACDF patients with and without Type II diabetes.

	No Type 2 Diabetes	Type 2 Diabetes	Significance
Length of stay, mean, in days	1.79 (Std. deviation 2.3)	1.99 (Std. deviation 2.6)	*p* < 0.001
Total charges, mean, in USD	67,998 (Std. deviation 56191)	71,884 (Std. deviation 55528)	*p* = 0.004

**Table 5:** Comparison of select postoperative complications in propensity score-matched single-level ACDF patients with and without type II diabetes.

Parameter	No Type 2 Diabetes	Type 2 Diabetes	Significance
DVT (%)	0.1	0.1	*p* = 0.157
Blood Loss Anemia (%)	20.1	20.5	*p* = 0.357

## Data Availability

Original contributions presented in this study are included in the article/Supplementary Materials; further inquiries can be directed to the corresponding author.

## Supplementary Materials

The following supporting information can be downloaded at: https://www.mdpi.com/article/10.3390/jcm14093039/s1.

## Abbreviations

ACDF	Anterior Cervical Discectomy and Fusion;
CI	Confidence Interval;
CSF	Cerebrospinal Fluid;
CVA	Cerebrovascular Accident;
DVT	Deep Vein Thrombosis;
HbA1c	Hemoglobin A1c;
HCUP	Healthcare Cost and Utilization Project;
HMO	Health Maintenance Organization;
ICD-10	International Classification of Diseases, 10th Revision;
IDDM	Insulin-Dependent Diabetes Mellitus;
LOS	Length of Stay;
NIDDM	Non-Insulin Dependent Diabetes Mellitus;
NIS	Nationwide Inpatient Sample;
OGTT	Oral Glucose Tolerance Test;
PSM	Propensity Score Matching;
RR	Risk Ratio;
SPSS	Statistical Package for the Social Sciences;
T2DM	Type 2 Diabetes Mellitus;
UTI	Urinary Tract Infection;
WHO	World Health Organization.
