# Supplementary material for: The Impact of Diabetes on Outcomes in Anterior Cervical Discectomy and Fusion (ACDF)
Source: J Clin Med. 2025 Apr 28;14(9):3039. doi: 10.3390/jcm14093039 (PMC12073059; doi:10.3390/jcm14093039)
Supplement: Supplementary file 1 [file jcm-14-03039-s001.zip › Table S2.pdf]

Condition	ICD-10 Code(s)
Urinary Tract Infection (UTI)	N39.0
Blood Loss Anemia	D62
Deep Vein Thrombosis (DVT)	I82.4
Dysphagia	R13.10, R13.11, R13.12
Horner Syndrome	H57.01, H57.02, H57.03
Cervical Spinal Cord Injury	S14.0XXA, S14.1XXA
